# Longitudinal dynamics of symptom networks in patients with differentiated thyroid cancer undergoing radioactive iodine therapy: a prospective cohort study

**DOI:** 10.3389/fonc.2026.1776771

**Published:** 2026-04-30

**Authors:** Juan Deng, Qin Deng, Li Zhang

**Affiliations:** 1 Department of Breast and Thyroid Surgery, The Second Affiliated Hospital of Chongqing Medical University, Chongqing, China; 2 School of Nursing, Chongqing Medical University, Chongqing, China

**Keywords:** bridge centrality, differentiated thyroid cancer, longitudinal study, psychological distress, radioactive iodine therapy, symptom network analysis

## Abstract

**Background:**

Patients with differentiated thyroid cancer (DTC) undergoing radioactive iodine (RAI) therapy experience concurrent physical and psychological symptoms, yet the dynamic interplay among these symptoms remains incompletely characterized.

**Objective:**

To characterize longitudinal changes in symptom network structure during RAI therapy and identify core and bridge symptoms across treatment phases.

**Methods:**

This prospective cohort study enrolled 520 DTC patients receiving RAI therapy. Symptoms were assessed at three timepoints: pre-treatment (T0), 48 hours post-treatment during radiation isolation (T1), and one week after discharge (T2). Gaussian graphical models were estimated using graphical LASSO with extended Bayesian information criterion. Strength centrality, bridge centrality, and predictability were calculated. Network comparison tests examined structural differences across timepoints.

**Results:**

Network structure differed significantly across timepoints (T0-T1: M = 0.306, P = 0.001; T0-T2: M = 0.347, P = 0.001), while global strength remained stable (P = 0.124-0.582). Psychological distress consistently exhibited the highest strength centrality (T0: 1.405; T1: 1.473; T2: 1.640) and bridge strength (T0: 0.755; T1: 0.767; T2: 0.976). Throat/mouth symptoms emerged as a critical bridge connecting physical and psychological symptom clusters after RAI (bridge strength: T0 = 0.107; T1 = 1.017; T2 = 1.016). Predictability of treatment-related physical symptoms increased substantially from near-zero at T0 to high levels at T1-T2 (e.g., throat/mouth symptoms: 0.064 to 0.782), indicating rapid network integration following treatment.

**Conclusions:**

RAI therapy is associated with substantial network reorganization rather than simple symptom accumulation. Psychological distress maintains central importance throughout, with increasing prominence post-treatment. Throat/mouth symptoms serve as critical bridges between physical and psychological domains. These findings suggest that the treatment-to-surveillance transition may represent an important window for psychological intervention, and that bridge symptoms such as throat/mouth discomfort merit further investigation as candidate targets for improving symptom management in DTC patients.

## 1 Introduction

Thyroid cancer is frequently characterized as “the good cancer,” yet patients’ lived experiences suggest otherwise: “there is no such thing as a good cancer” (1). Despite 10-year survival rates exceeding 97% for differentiated thyroid cancer (DTC) (2), nearly 30% of patients report moderate-to-severe psychological distress, significantly higher than patients with prostate cancer (14.9%) or bladder cancer (16.4%) who have comparable prognoses (3). Radioactive iodine (RAI) therapy, a cornerstone of post-surgical management for DTC (2), imposes unique stressors: thyroid hormone withdrawal induces hypothyroid symptoms before treatment, radiation isolation requires 2–3 days of solitary confinement, and patients subsequently face prolonged uncertainty awaiting treatment response evaluation (4–6). Throughout this process, patients simultaneously experience physical symptoms (salivary gland swelling, taste alterations, nausea) and psychological symptoms (anxiety, fear of cancer recurrence, loneliness) (7–9). This concurrent burden of multiple symptoms may amplify patients’ overall distress and compromise their quality of life during and after treatment. However, the dynamic interplay among these symptoms and whether specific symptoms drive overall symptom burden remain incompletely characterized.

Traditional symptom research treats individual symptoms as independent entities, failing to capture their dynamic interactions. Symptom cluster approaches recognize co-occurrence patterns but cannot delineate direct associations or transmission pathways among symptoms (10). Network analysis, originating from psychopathology research, offers a novel framework for understanding complex symptom relationships (11, 12). This approach conceptualizes symptoms as nodes and their associations as edges, using centrality indices to identify symptoms most connected to others, and bridge centrality to detect symptoms linking different symptom clusters (13). Such bridge symptoms have been proposed as potential intervention targets for attenuating symptom spread across domains (13, 14). Network analysis has been increasingly applied in oncology research. A recent scoping review synthesized 32 cancer symptom network studies, though most (66%) employed cross-sectional designs (15). Limited research has applied this approach to thyroid cancer populations. De Rooij et al. (16) included 190 thyroid cancer survivors and identified fatigue as the most central symptom; however, their cross-sectional design focused on the survivorship phase rather than the acute treatment period.

Against this background, we employed longitudinal network analysis to assess symptom networks at three critical timepoints during RAI therapy. At pre-treatment (T0), patients experience hypothyroid symptoms but have not yet received radiation exposure, representing baseline symptom patterns. At 48 hours post-treatment (T1), patients have just completed radiation isolation and are experiencing peak physical side effects superimposed on psychological stress. At one week post-discharge (T2), patients have returned home and entered a transitional phase of recovery and treatment response monitoring. This design captures dynamic network changes during the acute treatment period rather than merely obtaining a static snapshot at a single timepoint.

We aimed to compare symptom network structures across three timepoints, identify symptoms consistently occupying central positions, and explore the dynamic emergence of bridge symptoms during treatment. We hypothesized that RAI therapy would trigger significant network reorganization, with strengthened connections between physical and psychological symptoms and emergence of new bridge symptoms linking different symptom clusters.

To our knowledge, this study represents one of the first longitudinal investigations of symptom network dynamics in DTC patients during RAI therapy. Our findings offer a network perspective for understanding the symptom experiences of this “good cancer” population and may guide the development of symptom management strategies that account for symptom interconnection patterns.

## 2 Materials and methods

### 2.1 Study design and participants

This prospective longitudinal observational study was conducted at the Second Affiliated Hospital of Chongqing Medical University, from February 2024 to October 2025. Consecutive sampling was employed to recruit patients meeting the following inclusion criteria: (1) age ≥18 years; (2) pathologically confirmed DTC (papillary or follicular); (3) prior total or near-total thyroidectomy; (4) scheduled for first or repeat RAI therapy; and (5) sufficient literacy to complete Chinese-language questionnaires. Exclusion criteria included: (1) severe psychiatric illness requiring ongoing treatment; (2) concurrent malignancy; and (3) inability to complete follow-up assessments. Sample size was determined based on network analysis methodological recommendations suggesting approximately 20 participants per node for stable network estimation (17). With 20 symptom nodes, the target minimum sample was 400 participants.

Patient recruitment at the Department of Nuclear Medicine of the Second Affiliated Hospital of Chongqing Medical University took place between February 2024 and August 2025, with final T2 follow-up assessments completed by October 2025. Eligibility was assessed prior to RAI administration. The participant flow is detailed in the Results section and **Supplementary Figure 1**. Attrition analysis comparing completers and non-completers is presented in **Supplementary Table 1**.

### 2.2 Data collection

Symptom assessments were conducted at three timepoints: T0 (within 24 hours before RAI administration), T1 (approximately 48 hours post-treatment, during radiation isolation), and T2 (approximately one week after discharge). This timeline was designed to capture distinct RAI treatment phases: T0 represents baseline assessment when patients are hypothyroid but have not received radiation; T1 targets the acute reaction period when patients are isolated and experiencing peak physical symptoms concurrent with psychological stress; T2 assesses early recovery when patients have passed the acute phase, returned home, and entered a transitional period of awaiting treatment response while readjusting psychologically. T0 assessments were conducted in person by trained research nurses. Due to radiation isolation requirements, T1 assessments were completed via electronic questionnaire (Wenjuanxing platform). T2 assessments used telephone-assisted electronic questionnaires. Sociodemographic and clinical data were extracted from medical records at baseline.

### 2.3 Instruments

#### 2.3.1 Thyroid cancer-specific quality of life questionnaire

Disease-related symptoms were assessed using THYCA-QoL, a validated thyroid cancer-specific quality of life instrument (18, 19) with established use in Asian populations (20, 21). The questionnaire contains 24 items across seven multi-item scales: neuromuscular symptoms (2 items), sensory disturbances (1 item), voice problems (3 items), concentration difficulties (3 items), sympathetic symptoms (2 items), throat/mouth symptoms (3 items), and psychological distress (4 items); plus six single items: scar concerns, feeling cold, tingling sensations, weight changes, headache, and decreased sexual interest. Items are rated on 4-point Likert scales (1=not at all to 4=very much), with higher scores indicating greater symptom burden. We used the Chinese version with established reliability and validity in mainland Chinese DTC patients (6). For network analysis, scale scores were summed to form 13 THYCA-QoL-related nodes.

#### 2.3.2 Fear of cancer recurrence inventory-short form

Fear of cancer recurrence was measured using the FCRI severity subscale (22), comprising 9 items rated from 0 (never) to 4 (always), yielding total scores of 0–36 with higher scores indicating greater fear. The Chinese FCRI-SF has demonstrated satisfactory psychometric properties in Chinese cancer patients (23, 24). The FCRI-SF total score served as a single network node (FCR).

#### 2.3.3 UCLA loneliness scale-3 item version

Loneliness was assessed using UCLA-3 (25, 26), derived from the UCLA Loneliness Scale Version 3 (27). Three items assess feelings of lacking companionship, feeling left out, and feeling isolated, each rated from 1 (hardly ever) to 3 (often), yielding total scores of 3-9. The UCLA-3 total score served as a single network node (Lon).

#### 2.3.4 RAI treatment-related symptom assessment

Existing thyroid cancer-specific instruments such as THYCA-QoL were designed primarily for long-term survivors and do not adequately capture acute RAI treatment-related symptoms. Following established principles for developing disease-specific modules of cancer symptom assessment tools (28), we conducted a literature review to identify RAI treatment-related adverse effects and consulted clinical experts to select symptoms meeting the following criteria: (1) relatively high incidence reported in the literature; (2) clear pathophysiological basis; and (3) no conceptual overlap with existing THYCA-QoL items. Five items were identified: (1) salivary gland symptoms (parotid swelling or pain), as salivary glands highly express the sodium-iodide symporter (NIS) and actively concentrate RAI, causing radiation damage, with reported incidence of approximately 30% (29); (2) neck symptoms (neck swelling, pain, or discomfort), caused by radiation thyroiditis, with reported incidence of approximately 47% (30, 31); (3) taste alterations (dysgeusia or unusual taste), as taste bud cells also express NIS, with reported incidence of 10-16% (32, 33); (4) nausea, as RAI is excreted via the gastrointestinal tract and can irritate gastric mucosa, with nausea occurring in approximately 40% and overall gastrointestinal complaints in up to 67% of patients (31, 32); and (5) radiation worry (concern about radiation exposure to non-target body parts), a psychological stressor unique to RAI therapy. Each item was rated on a 0–10 numerical rating scale (NRS), which is widely used in oncology symptom assessment instruments due to its sensitivity and cross-study comparability (28). Content validity of the five items was reviewed by a panel of three clinical experts (two nuclear medicine physicians and one oncology nurse specialist) who confirmed the clinical relevance and wording clarity of each item. Because each item assesses a distinct symptom construct and enters the network as a separate node, these items do not constitute a unidimensional scale, and internal consistency analysis is not applicable. Zero-order inter-item correlations were examined across timepoints to evaluate construct distinctiveness (**Supplementary Table 8**).

### 2.4 Bias control

Several measures were implemented to minimize potential bias. Selection bias was reduced through consecutive sampling of all eligible patients during the study period. To minimize information bias, standardized validated questionnaires with established psychometric properties were used, and research nurses received uniform training before data collection. The electronic questionnaire system (Wenjuanxing) automatically checked for item completeness, prompting participants to complete any missed items before submission. This design minimized item-level missingness. To assess potential attrition bias, baseline characteristics were compared between completers and non-completers (**Supplementary Table 1**).

### 2.5 Statistical analysis

Descriptive statistics were calculated for sociodemographic and clinical variables. Normality was assessed using Shapiro-Wilk tests. Given non-normal distributions, continuous variables are presented as median and interquartile range (IQR); categorical variables as frequencies and percentages. Mann-Whitney U tests compared continuous variables; chi-square or Fisher’s exact tests compared categorical variables. For the final analytic sample (n=520), item-level missing data were minimal (<1% for any single item) due to the electronic questionnaire system’s completeness checks. Participants with incomplete questionnaire data (missing >20% of items on any scale) were excluded during the data cleaning phase and counted among those lost to follow-up. No imputation was performed given the negligible missing rate in the analyzed sample. Descriptive analyses were performed using SPSS Statistics version 29 (IBM Corporation, USA).

### 2.6 Network estimation

We estimated Gaussian graphical models at each timepoint employing the graphical LASSO algorithm with EBIC-based model selection (34). The EBIC hyperparameter γ was set to 0.5 to balance sensitivity and specificity. Networks comprised 20 symptom nodes organized into three clusters: THYCA physical symptoms (12 nodes: neuromuscular [NM], sensory [Sen], voice [Voi], concentration [Con], sympathetic [Sym], throat/mouth [TM], scar [Sca], cold [Chi], tingling [Tin], weight [Wei], headache [Hea], sexual interest [Sex]), psychological symptoms (4 nodes: psychological distress [Psy], fear of recurrence [FCR], loneliness [Lon], radiation worry [RW]), and RAI physical symptoms (4 nodes: salivary [Sal], neck [Nec], taste [Tas], nausea [Nau]). Edge weights represent partial correlations between symptoms after controlling for all other symptoms in the network. As networks were estimated cross-sectionally at each timepoint, edges reflect concurrent associations and do not imply temporal or causal directionality between symptoms.

### 2.7 Centrality and bridge centrality

Strength centrality, computed as the sum of absolute edge weights connected to each node, quantifies the total connectivity of a symptom regardless of edge sign (35). We also computed expected influence, which retains the sign of edge weights and thereby distinguishes nodes that primarily activate other symptoms (positive edges) from those with mixed or inhibitory associations (36). We calculated bridge strength centrality, defined as the summed edge weights linking a node to nodes in other clusters, to identify symptoms bridging different clusters (13).

### 2.8 Predictability

We estimated node predictability (R²) to quantify the variance proportion in each symptom accounted for by its network neighbors (37). Values range from 0 to 1, with higher values indicating greater influence from other symptoms in the network.

### 2.9 Network comparison

We employed network comparison tests (NCT) to examine differences in network structure and global strength across timepoints (38). This permutation-based method (1000 permutations) tests whether observed differences exceed chance expectations. Network structure invariance tests whether edge weight patterns differ between networks; global strength invariance tests whether overall connectivity differs.

### 2.10 Stability and accuracy

Network accuracy was evaluated via nonparametric bootstrapping with 1000 iterations to derive 95% confidence intervals for edge weights. Centrality stability was evaluated using case-dropping bootstrap, yielding correlation stability coefficients (CS-coefficients) representing the maximum proportion of cases that can be dropped while maintaining at least 0.7 correlation with original centrality indices. Following established guidelines, CS-coefficients of 0.50 or above indicate good stability, while values of 0.25 or above suggest acceptable stability (17).

Network analyses were conducted in R version 4.5.1. Network estimation and visualization used the qgraph package (39). Centrality indices and stability analyses used the bootnet package (17). Bridge centrality was calculated using the networktools package (13). Node predictability was estimated using the mgm package (37). Network comparisons used the NetworkComparisonTest package (38). Statistical significance was set at P<0.05 (two-tailed).

## 3 Results

### 3.1 Participant characteristics

Of 691 patients assessed for eligibility, 119 were excluded (85 not meeting inclusion criteria; 34 declined to participate). The remaining 572 patients were enrolled and completed T0 assessment. During follow-up, 33 participants were excluded before T1 due to incomplete questionnaire data (n=21), data inconsistency (n=7; i.e., identical responses across all items on a given scale, suggesting inattentive responding), or withdrawal of consent (n=5), leaving 539 participants who completed T1 assessment. Subsequently, 19 participants were lost before T2 due to inability to contact (n=9), incomplete questionnaire data (n=4), refusal to continue (n=4), or other reasons (n=2). Ultimately, 520 participants completed all three assessments (completion rate: 90.9%). The participant flow diagram is presented in **Supplementary Figure 1**.

Among the 520 completers, median age was 42.0 years (IQR = 13.0), 416 (80.0%) were female, 439 (84.4%) were married, and 438 (84.2%) were employed. Papillary carcinoma was diagnosed in 510 patients (98.1%), median maximum tumor diameter was 2.0 cm (IQR = 0.9), and 482 (92.7%) were classified as American Thyroid Association (ATA) intermediate-to-high risk. First RAI treatment was received by 490 patients (94.2%), with median dose of 120.0 mCi (IQR = 20.0) (**Table 1**).

**Table 1:** Baseline characteristics of completers (n=520).

Variables	Completers (n=520)
Sociodemographic characteristics	
Age, years, M (IQR)	42.0 (13.0)
Sex, n(%)	
Female	416 (80.0)
Male	104 (20.0)
Marital status, n(%)	
Married	439 (84.4)
Single	51 (9.8)
Divorced/Widowed	30 (5.8)
Education level, n(%)	
Bachelor’s degree or above	141 (27.1)
High school/Technical secondary	142 (27.3)
Associate degree	152 (29.2)
Junior high school or below	85 (16.3)
Employment status, n(%)	
Employed	438 (84.2)
Retired/Unemployed	62 (11.9)
Other	20 (3.8)
Monthly household income per capita, n(%)	
<3000 CNY	86 (16.5)
3000–5000 CNY	172 (33.1)
5000–10000 CNY	176 (33.8)
>10000 CNY	86 (16.5)
Medical insurance type, n(%)	
Urban-rural resident insurance	143 (27.5)
Urban employee insurance	348 (66.9)
Self-pay	29 (5.6)
Clinical characteristics	
Histological type, n(%)	
Papillary carcinoma	510 (98.1)
Follicular carcinoma	10 (1.9)
Maximum tumor diameter, cm, M (IQR)	2.0 (0.9)
Lymph node metastasis region, n(%)	
Lateral neck only	9 (1.7)
Central compartment only	362 (69.6)
Central + Lateral neck	149 (28.7)
Distant metastasis, n(%)	
No	518 (99.6)
Yes	2 (0.4)
ATA recurrence risk stratification, n(%)	
High risk	13 (2.5)
Intermediate-low risk	25 (4.8)
Intermediate-high risk	482 (92.7)
Comorbidity, n(%)	
None	376 (72.3)
Hypertension	80 (15.4)
Diabetes	28 (5.4)
Other	36 (6.9)
RAI treatment frequency, n(%)	
First time	490 (94.2)
Second time	27 (5.2)
Third time or more	3 (0.6)
RAI dose, mCi, M (IQR)	120.0 (20.0)

M, median; IQR, interquartile range; CNY, Chinese Yuan; ATA, American Thyroid Association.

Attrition analysis revealed that non-completers were older (median 47.5 vs. 42.0 years, P = 0.012), less likely to be employed (71.2% vs. 84.2%, P = 0.033), had higher comorbidity rates (48.1% vs. 27.7%, P = 0.015), and differed in ATA risk stratification distribution (P = 0.034). Groups did not differ significantly in sex, marital status, education, income, insurance type, histological type, tumor diameter, regional lymph node involvement, distant metastasis, RAI treatment frequency, or dose (all P>0.05) (**Supplementary Table 1**).

### 3.2 Symptom descriptive statistics

Symptom means and standard deviations across timepoints are presented in **Table 2**. At T0, psychological distress scored highest (7.93 ± 1.58), followed by fear of cancer recurrence (15.05 ± 3.03); RAI physical symptoms scored low (salivary symptoms: 0.72 ± 0.78; taste alterations: 0.64 ± 0.67; nausea: 0.48 ± 0.55). At T1, all RAI physical symptoms increased substantially: salivary symptoms 6.48 ± 2.22, taste alterations 6.69 ± 2.22, throat/mouth symptoms 7.39 ± 1.66, neck symptoms 5.77 ± 2.04, and nausea 5.55 ± 2.43. Psychological symptoms increased concurrently: fear of cancer recurrence 17.55 ± 3.10, loneliness 7.47 ± 1.54, and psychological distress 9.22 ± 1.84. At T2, most symptoms decreased from T1: salivary symptoms 3.01 ± 1.94, taste alterations 3.46 ± 2.02, and nausea 1.67 ± 1.32. Psychological distress (6.83 ± 1.38) and fear of cancer recurrence (12.41 ± 2.93) remained elevated above T0 levels.

**Table 2:** Descriptive statistics of symptoms across three timepoints (M ± SD).

Node	Cluster	Description	T0	T1	T2
NM	THYCA-Somatic	Neuromuscular	4.59 ± 1.23	4.22 ± 1.20	3.65 ± 1.05
Sen	THYCA-Somatic	Sensory	2.16 ± 0.86	2.03 ± 0.80	1.72 ± 0.69
Voi	THYCA-Somatic	Voice	4.57 ± 1.00	6.29 ± 1.46	5.12 ± 1.14
Con	THYCA-Somatic	Concentration	6.35 ± 1.49	6.14 ± 1.34	4.96 ± 1.12
Sym	THYCA-Somatic	Sympathetic	3.13 ± 0.89	3.90 ± 1.15	3.02 ± 0.82
TM	THYCA-Somatic	Throat/Mouth	4.59 ± 0.99	7.39 ± 1.66	5.56 ± 1.34
Sca	THYCA-Somatic	Scar	1.76 ± 0.70	1.75 ± 0.69	1.65 ± 0.66
Chi	THYCA-Somatic	Chilliness	2.42 ± 0.95	1.97 ± 0.76	1.69 ± 0.64
Tin	THYCA-Somatic	Tingling	2.18 ± 0.84	1.87 ± 0.72	1.68 ± 0.66
Wei	THYCA-Somatic	Weight	2.04 ± 0.84	1.82 ± 0.76	1.69 ± 0.66
Hea	THYCA-Somatic	Headache	1.57 ± 0.59	2.04 ± 0.80	1.59 ± 0.61
Sex	THYCA-Somatic	Sexual interest	2.43 ± 0.82	2.49 ± 0.79	2.21 ± 0.80
Psy	Psychological	Psychological distress	7.93 ± 1.58	9.22 ± 1.84	6.83 ± 1.38
FCR	Psychological	Fear of recurrence	15.05 ± 3.03	17.55 ± 3.10	12.41 ± 2.93
Lon	Psychological	Loneliness	4.78 ± 1.12	7.47 ± 1.54	5.64 ± 1.40
RW	Psychological	Radiation worry	4.60 ± 2.24	5.95 ± 2.09	3.18 ± 1.86
Sal	RAI-Somatic	Salivary symptoms	0.72 ± 0.78	6.48 ± 2.22	3.01 ± 1.94
Nec	RAI-Somatic	Neck symptoms	1.14 ± 1.05	5.77 ± 2.04	2.84 ± 1.74
Tas	RAI-Somatic	Taste changes	0.64 ± 0.67	6.69 ± 2.22	3.46 ± 2.02
Nau	RAI-Somatic	Gastrointestinal	0.48 ± 0.55	5.55 ± 2.43	1.67 ± 1.32

T0, Pre-treatment; T1, 48h post-treatment; T2, 1 week post-discharge.

THYCA-Somatic, thyroid cancer-specific somatic symptoms; RAI-Somatic, radioactive iodine treatment-specific somatic symptoms.

Zero-order correlations among the five RAI symptom items are presented in **Supplementary Table 8**. At T0, inter-item correlations were negligible (r range: −0.07 to 0.05). At T1, correlations among the four somatic items increased (r = 0.72–0.79); radiation worry showed moderate correlations with somatic items (r = 0.55–0.63). At T2, nausea correlations with other somatic items attenuated (r = 0.41–0.48) while salivary–neck–taste correlations remained high (r = 0.78–0.82).

### 3.3 Network structure

**Figure 1:**
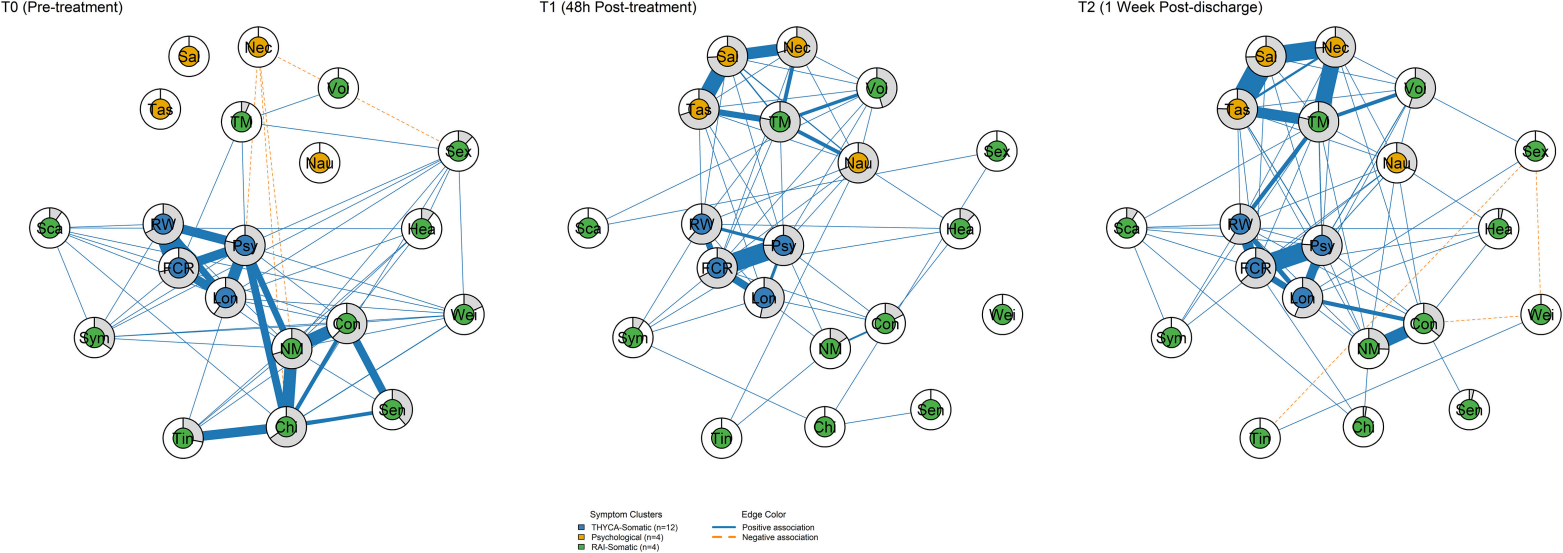
Symptom networks across three timepoints. Networks display 20 symptom nodes organized into three clusters: THYCA-Somatic (blue, n=12), Psychological (yellow, n=4), and RAI-Somatic (red, n=4). Edge thickness represents association strength; blue edges indicate positive associations, red edges indicate negative associations. T0 = pre-treatment; T1 = 48h post-treatment; T2 = 1 week post-discharge.

Symptom networks across three timepoints are displayed in **Figure 1**. Networks contained 20 symptom nodes organized into three predefined clusters: thyroid cancer physical symptoms (12 nodes), psychological symptoms (4 nodes), and RAI physical symptoms (4 nodes). At T0, dense interconnections were evident within the thyroid cancer physical symptom cluster and psychological symptom cluster, while the RAI physical symptom cluster showed sparse connections with other clusters. At T1, dense connections emerged within the RAI physical symptom cluster, and a strong edge formed between throat/mouth symptoms (TM) and psychological distress (Psy). The T2 network maintained the basic connectivity pattern of T1, with some edge weights attenuated.

### 3.4 Centrality analysis

**Figure 2:**
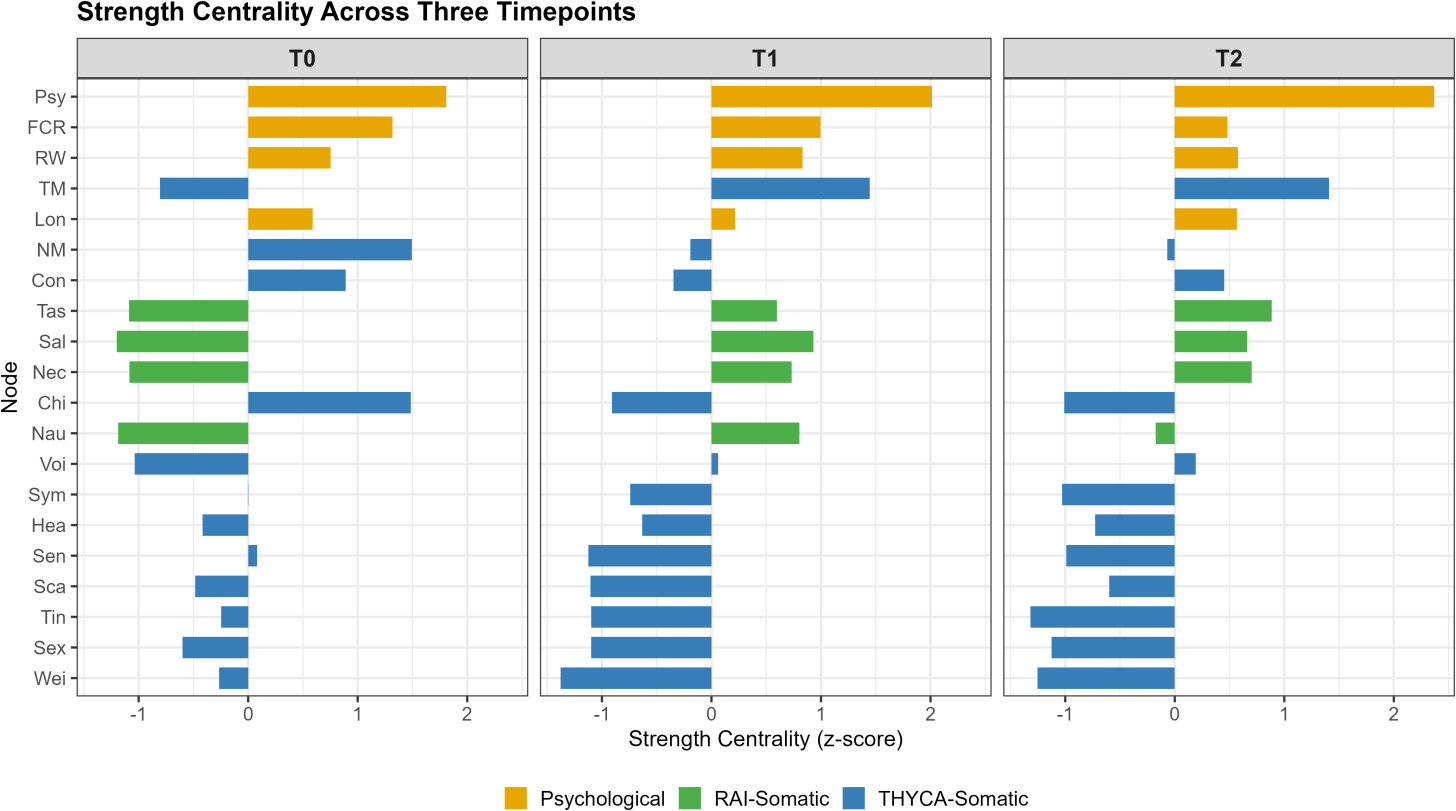
Strength centrality across three timepoints. Higher values indicate stronger overall connections with other symptoms in the network. Symptoms are ordered by mean strength centrality across timepoints.

Strength centrality values are displayed in **Figure 2** (numerical values in **Supplementary Table 2**). Psychological distress (Psy) exhibited the highest strength centrality across all three timepoints (T0: 1.405; T1: 1.473; T2: 1.640). Fear of cancer recurrence (FCR) strength centrality decreased from 1.175 at T0 to 0.839 at T2. Throat/mouth symptoms (TM) strength centrality increased from 0.185 at T0 to 1.231 at T1 and 1.233 at T2. Radiation worry (RW) strength centrality remained relatively stable across timepoints (0.880-0.969). Longitudinal trajectories of strength centrality are shown in **Supplementary Figure 4**.

### 3.5 Bridge centrality analysis

**Figure 3:**
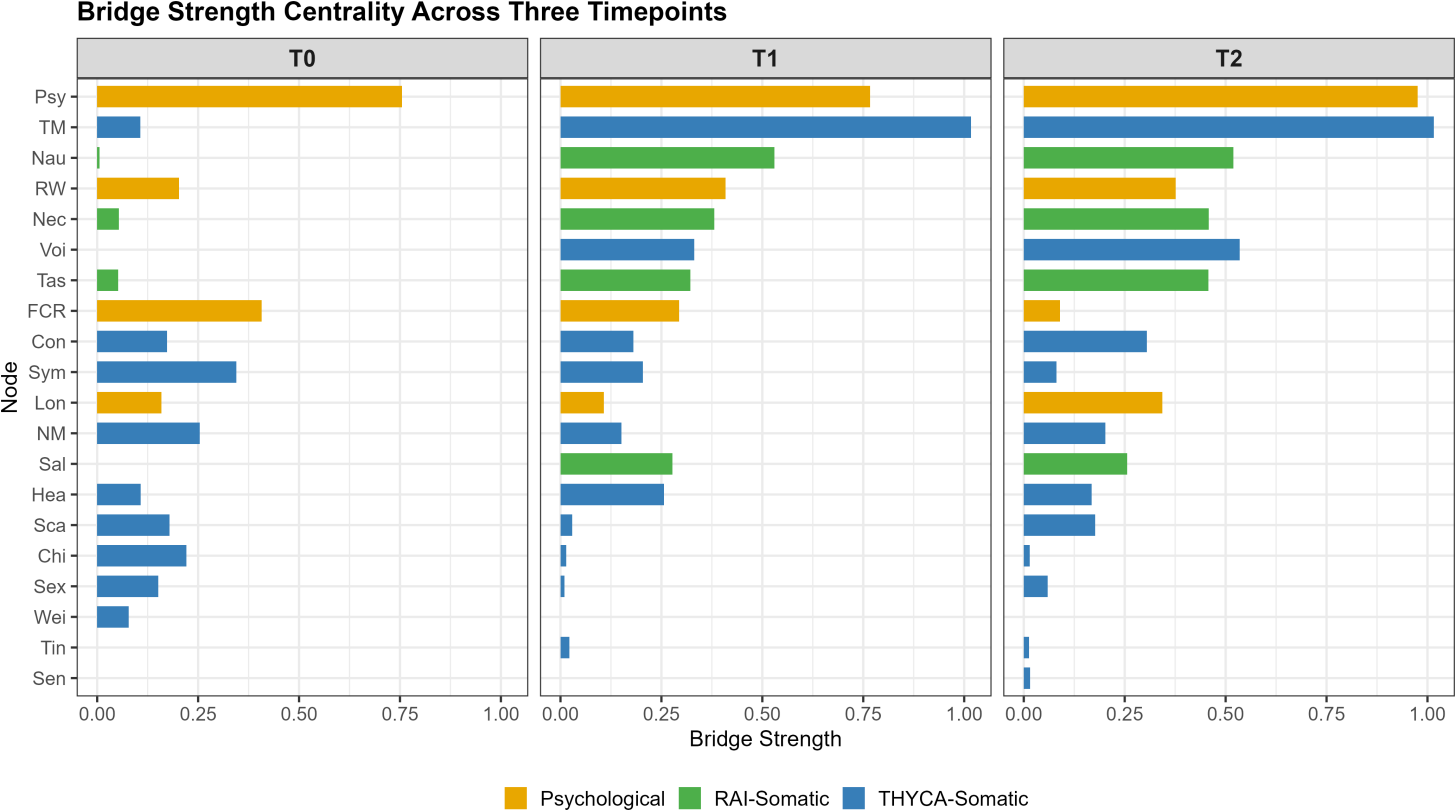
Bridge strength centrality across three timepoints. Higher values indicate stronger connections with symptoms in other clusters. Symptoms are colored by cluster membership.

Bridge strength centrality values are displayed in **Figure 3** (numerical values in **Supplementary Table 3**). Psychological distress (Psy) exhibited the highest bridge strength across all three timepoints (T0: 0.755; T1: 0.767; T2: 0.976). Throat/mouth symptoms (TM) bridge strength increased from 0.107 at T0 to 1.017 at T1 and 1.016 at T2. Nausea (Nau) bridge strength reached 0.530 at T1 and 0.519 at T2. Neck symptoms (Nec) bridge strength increased from 0.054 at T0 to 0.381 at T1 and 0.458 at T2. Longitudinal trajectories of bridge strength are shown in **Supplementary Figure 5**.

### 3.6 Predictability analysis

**Figure 4:**
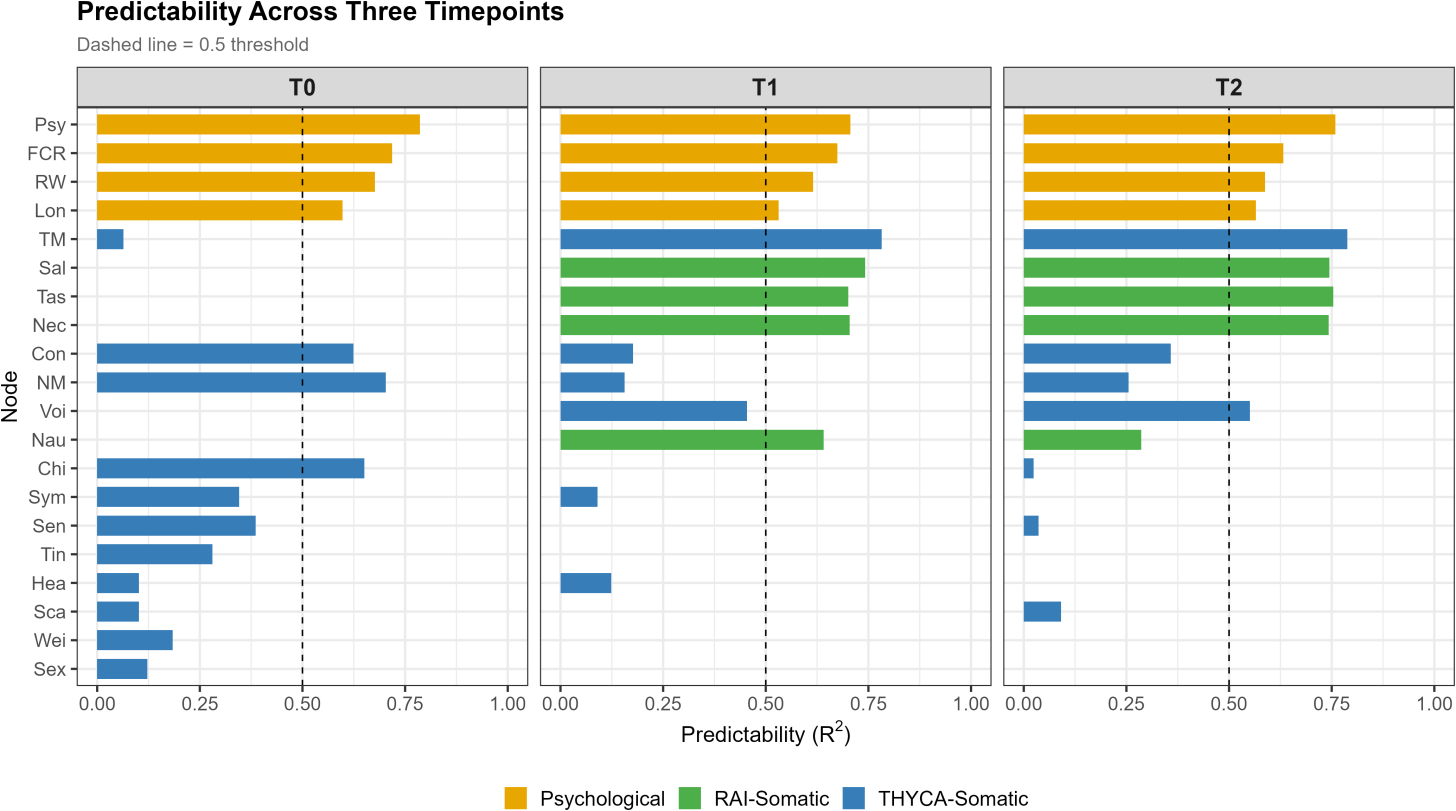
Predictability (R²) across three timepoints. Values represent the proportion of variance in each symptom explained by its network neighbors. Dashed line indicates R²=0.5 threshold.

Node predictability values are displayed in **Figure 4** (numerical values in **Supplementary Table 6**). Psychological symptom cluster nodes showed consistently high predictability across timepoints: psychological distress T0 = 0.786, T1 = 0.706, T2 = 0.759; fear of cancer recurrence T0 = 0.718, T1 = 0.674, T2 = 0.632. RAI physical symptom cluster nodes showed near-zero predictability at T0 but substantial increases at T1 and T2: salivary symptoms T1 = 0.742, T2 = 0.744; taste alterations T1 = 0.701, T2 = 0.754; neck symptoms T1 = 0.704, T2 = 0.743. Throat/mouth symptoms showed the largest change in predictability (T0: 0.064; T1: 0.782; T2: 0.788).

### 3.7 Network stability

Edge weight accuracy analyses are presented in **Supplementary Figure 2**; bootstrap 95% confidence intervals were narrow across all three timepoints. Centrality stability analyses are presented in **Supplementary Figure 3**; CS-coefficients for strength centrality and expected influence were 0.75 at all three timepoints (**Supplementary Table 7**).

### 3.8 Network comparison

Network comparison test results are presented in **Table 3**. Network structure differed significantly between T0-T1 (M = 0.306, 95% CI [0.095, 0.172], P = 0.001), T1-T2 (M = 0.169, 95% CI [0.086, 0.165], P = 0.021), and T0-T2 (M = 0.347, 95% CI [0.091, 0.170], P = 0.001). Global strength differences were not statistically significant: T0-T1 (ΔS=0.384, P = 0.304), T1-T2 (ΔS=0.194, P = 0.582), T0-T2 (ΔS=0.578, P = 0.124). Global strength values were T0 = 5.431, T1 = 5.815, T2 = 6.009.

**Table 3:** Network comparison test (NCT) results.

Comparison	Network Structure	Global Strength
	M [95% CI], P	ΔS [95% CI], P
T0 vs T1	0.306 [0.095, 0.172], **0.001**	0.384 [0.018, 0.819], 0.304
T1 vs T2	0.169 [0.086, 0.165], **0.021**	0.194 [0.008, 0.801], 0.582
T0 vs T2	0.347 [0.091, 0.170], **0.001**	0.578 [0.012, 0.805], 0.124

Global strength values: T0 = 5.431, T1 = 5.815, T2 = 6.009.

M, maximum absolute difference in edge weights between networks.

ΔS, difference in global strength (sum of absolute edge weights).

95% CI, 95% confidence interval based on permutation distribution (1000 iterations).

Bold P values indicate statistical significance (P<0.05).

## 4 Discussion

Using longitudinal network analysis, this study characterized symptom network dynamics during the acute phase of RAI therapy in DTC patients. Three principal findings merit discussion: network structure underwent significant reorganization across treatment phases while global strength remained relatively stable; psychological distress consistently occupied the central network position with increasing prominence post-treatment; and throat/mouth symptoms emerged as a critical bridge connecting physical and psychological symptom clusters following RAI. These results indicate that DTC patients undergoing RAI therapy experience not merely symptom accumulation and resolution, but dynamic network reorganization. Attending to symptom interconnection patterns, rather than focusing solely on individual symptom severity, may enable deeper understanding of patient symptom experiences and inform targeted intervention development.

### 4.1 Network reorganization with stable global strength

Network comparison tests revealed significant structural differences across timepoints (T0-T1: M = 0.306, P = 0.001; T0-T2: M = 0.347, P = 0.001), while global strength changes did not reach statistical significance (P = 0.124-0.582). This pattern suggests that RAI therapy was associated with different connection patterns among symptoms rather than simply higher or lower overall association strength. This observation aligns with network theory in psychopathology research, which posits that the specific configuration of symptom connections, rather than merely their aggregate intensity, shapes how symptoms co-occur (11, 40). Our findings extend this framework to the cancer treatment context, indicating that physical and psychological symptom associations similarly differ in their interconnection patterns across treatment phases. It should be noted that because each network was estimated cross-sectionally, edges within a given timepoint capture concurrent associations and do not indicate temporal or directional influence between symptoms. Likewise, the network comparison tests detect structural differences in edge weight patterns between timepoints but do not establish that one network causally evolved into another.

Although global strength differences did not reach significance, values showed an upward trend from T0 to T2 (5.431 to 6.009, approximately 10.6% increase). Given our sample size, this non-significant finding may reflect limited statistical power; future studies with larger samples could provide clarification. Moreover, global strength reflects only overall network connectivity density, whereas functional properties of symptom networks may depend more on specific connection patterns (i.e., which symptoms connect to which) rather than total connection quantity (40, 41). From this perspective, significant changes in network structure may carry greater clinical relevance than changes in global strength.

This finding holds potential implications for clinical practice. If symptom networks respond to treatment events through connection pattern reorganization rather than overall strength changes, it is plausible that attending to the distribution of symptom connections, in addition to reducing total symptom scores, could inform future intervention design (15, 42). This inference rests on observed network associations and would need to be tested in experimental intervention research before guiding clinical recommendations.

### 4.2 The central position of psychological distress

Despite being considered “the good cancer,” research indicates that psychological distress levels in thyroid cancer patients are not lower than in other cancer types. Gao et al. (43) found moderate psychological distress in DTC patients during transitional periods, closely associated with illness perception and fatigue. Network analysis has emerged as a valuable approach for understanding symptom interconnections in cancer populations (44–46). Psychological distress (Psy) consistently exhibited the highest strength centrality and bridge strength throughout the observation period, indicating extensive and close associations with other network symptoms. This finding aligns with prior cancer symptom network research. A recent systematic review examining network analysis in cancer populations confirmed that psychological symptoms, particularly anxiety, depression, and distress, are frequently identified as central and stably interconnected nodes across various cancer types and treatment stages (47). Zhu et al. (48) found that distress and sadness exhibited the highest strength centrality in cancer survivor multidimensional symptom networks, with distress showing the largest centrality value (rs = 9.18). Our observation of similar patterns in the DTC population suggests that the central role of psychological distress may generalize across cancer types and treatment phases.

Notably, de Rooij et al. (16) identified fatigue as the most central symptom in thyroid cancer survivors, whereas we found psychological distress occupying the central position. This discrepancy likely reflects different study periods: de Rooij et al. focused on survivorship when acute treatment stress has subsided and fatigue persists as a dominant symptom; our study focused on the acute RAI treatment period when patients experience significant psychological stress from radiation isolation and treatment outcome uncertainty, rendering psychological distress more prominent.

Of particular interest is the increasing trend in psychological distress centrality and bridge strength following RAI treatment (strength centrality: T0 = 1.405, T1 = 1.473, T2 = 1.640; bridge strength: T0 = 0.755, T1 = 0.767, T2 = 0.976). This finding suggests that completing RAI treatment did not bring anticipated psychological relief; rather, patients’ psychological burden may have increased. Lebel et al. (49) identified uncertainty and loss of control as core drivers of fear of cancer recurrence. After RAI completion, patients transition from active treatment to surveillance. During this transition, clear treatment goals give way to persistent worry about recurrence and anxious anticipation of test results. We speculate that this role transition from “active treatment recipient” to “passive surveillance subject” may partly explain the persistence or intensification of psychological distress, though this interpretation awaits empirical confirmation. Indeed, managing FCR has been identified as one of the most important unmet needs among cancer survivors, with high FCR related to increased healthcare utilization and lower quality of life (50).

Psychological distress showed extensive statistical associations with other symptoms in the network. This pattern is consistent with the theoretical premise that symptoms occupying central positions may be promising candidates for intervention, yet centrality derived from cross-sectional network estimation reflects the strength of concurrent associations rather than causal influence (14, 51). High centrality alone does not guarantee that intervening on a given node will improve downstream symptoms (52). Prior meta-analyses demonstrate that cognitive behavioral therapy and mindfulness interventions effectively reduce psychological distress in cancer patients (53, 54). The high centrality of psychological distress in our networks raises the hypothesis that such interventions might yield effects beyond the directly targeted symptom, but this hypothesis is derived from observational associations and awaits verification through trials designed to test network-level intervention effects. Additionally, the timing of psychological support warrants attention: the post-RAI transitional period may represent an important window for psychological intervention, helping patients adapt to the shift from treatment to surveillance and rebuild sense of life control.

### 4.3 The bridge role of throat/mouth symptoms

Throat/mouth symptoms (TM) showed markedly elevated bridge centrality following RAI (from 0.107 at T0 to 1.017 at T1), emerging as a key node connecting RAI physical symptoms and psychological symptom clusters. Jones et al. (13) proposed that bridge symptoms serve as conduits linking different symptom clusters, such that their associations span cluster boundaries. In simulation studies, deactivating bridge nodes reduced cross-cluster symptom co-activation, though empirical validation of this mechanism in clinical populations remains limited (36, 55). Our longitudinal observation of dynamic bridge symptom emergence suggests that treatment events can alter network bridging structure.

The biological basis for throat/mouth symptoms becoming a bridge node likely relates to RAI tissue distribution. Salivary glands possess iodine-concentrating capacity and are thus susceptible to radiation damage following RAI, causing xerostomia, taste alterations, and oral mucositis (29, 31). The question of why throat/mouth symptoms, rather than other RAI side effects such as nausea, became the critical bridge between physical and psychological symptoms warrants consideration. Bridge strength data show TM at T1 (1.017) substantially exceeded nausea (0.530). One possible explanation relates to the social dimensions of oral function. Eating is central to family and social gatherings, speech underlies occupational function, and taste contributes to daily life enjoyment. If RAI-induced oral mucositis, salivary dysfunction, and taste alterations disrupt these social functions, the resulting impact on social participation and quality of life may account for the stronger bridging to psychological symptoms. Head and neck cancer research similarly documents associations between swallowing and speech dysfunction with social isolation and psychological distress (56). In contrast, nausea primarily affects individual comfort with less direct impact on social function, potentially explaining its relatively limited bridging role. This interpretation is speculative and requires verification through mediation analysis or qualitative interviews.

From a clinical standpoint, the bridge position of throat/mouth symptoms highlights the relevance of proactive oral symptom management. Prior research indicates that salivary gland protective measures (adequate hydration, sour candy to stimulate saliva, mouth rinses) can partially alleviate RAI-related xerostomia (57, 58); oral care guidance and taste rehabilitation training may improve patients’ eating experience. Whether such interventions also carry downstream benefits for psychological well-being—as the bridging position of this node might suggest—remains an open question. Bridge centrality quantifies the strength of cross-cluster associations and does not confirm that targeting a bridge node will interrupt symptom activation across domains (51, 55). Intervention trials tracking network-level outcomes would be needed to test this possibility.

### 4.4 Dynamic changes in symptom predictability

Predictability analysis revealed an interesting pattern in RAI physical symptoms: near-zero predictability at T0 but marked increases at T1 and T2. According to Haslbeck and Waldorp (37), predictability (R²) reflects the degree to which a node is influenced by its network neighbors. Low predictability of RAI physical symptoms at T0 does not indicate complete absence of these symptoms but rather indicates lack of systematic association with other network symptoms before treatment. Patients may experience mild fatigue or discomfort during post-surgical thyroid-stimulating hormone (TSH) suppression, but these symptoms remain relatively independent without tight connections to psychological symptoms. Following RAI, predictability of these symptoms increased dramatically (e.g., TM from 0.064 to 0.782), suggesting rapid integration into the symptom network with formation of tight associations with other symptoms. This pattern carries theoretical significance: RAI physical symptoms, though initially triggered by external factors (direct radioiodine effects), may become closely embedded within the broader symptom network once they emerge. This network integration may contribute to symptom persistence in some patients.

This finding has practical relevance for symptom management timing. Prior to RAI treatment, physical symptoms remain relatively isolated and may be more amenable to intervention. Once these symptoms become embedded in a densely connected network, their strong concurrent associations with other symptoms may make them more difficult to treat in isolation (11, 40). Preventive interventions initiated before or shortly after RAI administration may therefore be more effective than reactive management after dense symptom associations have formed. Future research should explore whether early intervention can prevent or attenuate this network integration process.

### 4.5 Limitations

Several limitations should be acknowledged. First, the single-center design ensured standardized RAI protocols and consistent data collection but may limit generalizability to other clinical settings; multi-center validation is needed. Second, the three timepoints were selected to capture clinically distinct treatment phases yet cannot fully delineate symptom network evolution; ecological momentary assessment designs could provide finer temporal resolution in future studies (59, 60). Third, the five RAI treatment-related symptom items were developed for this study and have not undergone formal psychometric validation. Although content validity was supported by expert review and items captured independent constructs at baseline (**Supplementary Table 8**), the somatic items showed elevated correlations following RAI, and content proximity with the THYCA-QoL throat/mouth scale cannot be excluded. The GGM’s partial correlation framework mitigates this concern analytically, but formal discriminant validity evaluation in independent samples is warranted. Fourth, networks were estimated cross-sectionally at each timepoint and capture concurrent associations rather than causal or temporal relationships; intervention studies are needed to test whether addressing central or bridge symptoms improves overall symptom burden. Regarding generalizability, although attrition was low (9.1%) and groups did not differ in sex, education, tumor features, or RAI dose, non-completers were older, less frequently employed, and had more comorbidities, which may be associated with higher somatic symptom burden. Our findings may therefore be most applicable to relatively young, employed DTC patients with low comorbidity burden. The predominantly female (80%) and papillary carcinoma (98.1%) sample reflects DTC epidemiological characteristics but may limit applicability to populations with different demographic or clinical profiles; cross-cultural validation is warranted.

### 4.6 Conclusions

In DTC patients undergoing RAI therapy, symptom networks demonstrate significant structural reorganization while maintaining stable global connectivity. Psychological distress maintains a central position throughout, and its network influence increases following treatment. Throat/mouth symptoms emerge as critical bridge nodes linking physical and psychological symptom clusters. These findings suggest that the treatment-to-surveillance transition may represent an important window for psychological intervention, and that bridge symptoms such as throat/mouth discomfort merit further investigation as candidate targets for improving symptom management in DTC patients. Clinical symptom management should attend not only to individual symptom severity but also to symptom interconnection patterns within the network.

## Data Availability

Due to ethical restrictions and the sensitive nature of the data (patient questionnaire responses and personal information), the datasets generated and analyzed during this study are not publicly available. Anonymized data may be available from the corresponding author upon reasonable request, subject to approval from the Ethics Committee of Chongqing Medical University. Requests to access the datasets should be directed to Li Zhang, zhangli@hospital.cqmu.edu.cn.

## Supplementary material

Supplementary Table 1 Comparison of baseline characteristics between completers and non-completers.

Supplementary Table 2 Strength centrality values across three timepoints.

Supplementary Table 3 Bridge strength centrality values across three timepoints.

Supplementary Table 4 Expected influence centrality values across three timepoints.

Supplementary Table 5 Bridge expected influence centrality values across three timepoints.

Supplementary Table 6 Predictability (R²) values across three timepoints.

Supplementary Table 7 Centrality stability coefficients (CS-coefficients).

Supplementary Table 8 Zero-order Pearson correlations among five RAI treatment-related symptom items and THYCA-QoL throat/mouth scale across three timepoints.

Supplementary Figure 1 Participant flow diagram showing enrollment, follow-up, and attrition at each assessment timepoint.

Supplementary Figure 2 Edge weight accuracy (bootstrap 95% CI) at T0, T1, and T2.

Supplementary Figure 3 Centrality stability (case-dropping bootstrap) at T0, T1, and T2.

Supplementary Figure 4 Strength centrality trajectories across timepoints.

Supplementary Figure 5 Bridge strength centrality trajectories across timepoints.
