# Supplementary material for: Longitudinal dynamics of symptom networks in patients with differentiated thyroid cancer undergoing radioactive iodine therapy: a prospective cohort study
Source: Front Oncol. 2026 Apr 30;16:1776771. doi: 10.3389/fonc.2026.1776771 (PMC13171378; doi:10.3389/fonc.2026.1776771)
Supplement: Supplementary Table 1 — Comparison of baseline characteristics between completers and non-completers. [file Table1.docx]

**Supplementary Material**

**Longitudinal Dynamics of Symptom Networks in Patients with Differentiated Thyroid Cancer Undergoing Radioactive Iodine Therapy: A Prospective Cohort Study**

**Table S1. Comparison of baseline characteristics between completers and non-completers**

| **Variables** | **Completers (n=520)** | **Non-completers (n=52)** | **P value** |
| --- | --- | --- | --- |
| **Sociodemographic characteristics** |  |  |  |
| Age, years, M (IQR) | 42.0 (13.0) | 47.5 (17.0) | **0.012** |
| Sex, n(%) |  |  | 0.856† |
| Female | 416 (80.0) | 41 (78.8) |  |
| Male | 104 (20.0) | 11 (21.2) |  |
| Marital status, n(%) |  |  | 0.500† |
| Married | 439 (84.4) | 45 (86.5) |  |
| Single | 51 (9.8) | 6 (11.5) |  |
| Divorced/Widowed | 30 (5.8) | 1 (1.9) |  |
| Education level, n(%) |  |  | 0.395† |
| Bachelor's degree or above | 141 (27.1) | 11 (21.2) |  |
| High school/Technical secondary | 142 (27.3) | 15 (28.8) |  |
| Associate degree | 152 (29.2) | 13 (25.0) |  |
| Junior high school or below | 85 (16.3) | 13 (25.0) |  |
| Employment status, n(%) |  |  | **0.033†** |
| Employed | 438 (84.2) | 37 (71.2) |  |
| Retired/Unemployed | 62 (11.9) | 13 (25.0) |  |
| Other | 20 (3.8) | 2 (3.8) |  |
| Monthly household income per capita, n(%) |  |  | 0.965† |
| <3000 CNY | 86 (16.5) | 10 (19.2) |  |
| 3000-5000 CNY | 172 (33.1) | 17 (32.7) |  |
| 5000-10000 CNY | 176 (33.8) | 17 (32.7) |  |
| >10000 CNY | 86 (16.5) | 8 (15.4) |  |
| Medical insurance type, n(%) |  |  | 0.227† |
| Urban-rural resident insurance | 143 (27.5) | 19 (36.5) |  |
| Urban employee insurance | 348 (66.9) | 29 (55.8) |  |
| Self-pay | 29 (5.6) | 4 (7.7) |  |
| **Clinical characteristics** |  |  |  |
| Histological type, n(%) |  |  | 0.611† |
| Papillary carcinoma | 510 (98.1) | 52 (100.0) |  |
| Follicular carcinoma | 10 (1.9) | 0 (0.0) |  |
| Maximum tumor diameter, cm, M (IQR) | 2.0 (0.9) | 1.9 (0.9) | 0.212 |
| Lymph node metastasis region, n(%) |  |  | 0.590† |
| Lateral neck only | 9 (1.7) | 1 (1.9) |  |
| Central compartment only | 362 (69.6) | 39 (75.0) |  |
| Central + Lateral neck | 149 (28.7) | 12 (23.1) |  |
| Distant metastasis, n(%) |  |  | 1.000† |
| No | 518 (99.6) | 52 (100.0) |  |
| Yes | 2 (0.4) | 0 (0.0) |  |
| ATA recurrence risk stratification, n(%) |  |  | **0.034†** |
| High risk | 13 (2.5) | 0 (0.0) |  |
| Intermediate-low risk | 25 (4.8) | 7 (13.5) |  |
| Intermediate-high risk | 482 (92.7) | 45 (86.5) |  |
| Comorbidity, n(%) |  |  | **0.015†** |
| None | 376 (72.3) | 27 (51.9) |  |
| Hypertension | 80 (15.4) | 14 (26.9) |  |
| Diabetes | 28 (5.4) | 4 (7.7) |  |
| Other | 36 (6.9) | 7 (13.5) |  |
| RAI treatment frequency, n(%) |  |  | 1.000† |
| First time | 490 (94.2) | 50 (96.2) |  |
| Second time | 27 (5.2) | 2 (3.8) |  |
| Third time or more | 3 (0.6) | 0 (0.0) |  |
| RAI dose, mCi, M (IQR) | 120.0 (20.0) | 100.0 (20.0) | 0.078 |

*M: median; IQR: interquartile range; CNY: Chinese Yuan; ATA: American Thyroid Association; RAI: radioactive iodine.*

*†Fisher's exact test (Fisher-Freeman-Halton test for R×C tables). Continuous variables were compared using Mann-Whitney U test.*

*Bold P values indicate statistical significance (P<0.05).*

**Table S2. Strength centrality across three timepoints**

| **Node** | **Cluster** | **T0** | **T1** | **T2** |
| --- | --- | --- | --- | --- |
| NM | THYCA-Somatic | 1.258 | 0.531 | 0.606 |
| Sen | THYCA-Somatic | 0.598 | 0.134 | 0.216 |
| Voi | THYCA-Somatic | 0.076 | 0.640 | 0.716 |
| Con | THYCA-Somatic | 0.976 | 0.466 | 0.827 |
| Sym | THYCA-Somatic | 0.559 | 0.297 | 0.199 |
| TM | THYCA-Somatic | 0.185 | 1.231 | 1.233 |
| Sca | THYCA-Somatic | 0.335 | 0.142 | 0.382 |
| Chi | THYCA-Somatic | 1.254 | 0.226 | 0.208 |
| Tin | THYCA-Somatic | 0.446 | 0.145 | 0.077 |
| Wei | THYCA-Somatic | 0.436 | 0.025 | 0.104 |
| Hea | THYCA-Somatic | 0.366 | 0.343 | 0.327 |
| Sex | THYCA-Somatic | 0.280 | 0.145 | 0.158 |
| Psy | Psychological | 1.405 | 1.473 | 1.640 |
| FCR | Psychological | 1.175 | 1.039 | 0.839 |
| Lon | Psychological | 0.835 | 0.706 | 0.876 |
| RW | Psychological | 0.911 | 0.969 | 0.880 |
| Sal | RAI-Somatic | 0.000 | 1.011 | 0.915 |
| Nec | RAI-Somatic | 0.054 | 0.926 | 0.933 |
| Tas | RAI-Somatic | 0.052 | 0.869 | 1.011 |
| Nau | RAI-Somatic | 0.006 | 0.957 | 0.562 |

*Strength centrality represents the sum of absolute edge weights connected to each node.*

*T0: Pre-treatment; T1: 48h post-treatment; T2: 1 week post-discharge.*

**Table S3. Bridge strength centrality across three timepoints**

| **Node** | **Cluster** | **T0** | **T1** | **T2** |
| --- | --- | --- | --- | --- |
| NM | THYCA-Somatic | 0.254 | 0.151 | 0.202 |
| Sen | THYCA-Somatic | 0.000 | 0.000 | 0.016 |
| Voi | THYCA-Somatic | 0.000 | 0.331 | 0.535 |
| Con | THYCA-Somatic | 0.173 | 0.181 | 0.305 |
| Sym | THYCA-Somatic | 0.345 | 0.204 | 0.081 |
| TM | THYCA-Somatic | 0.107 | 1.017 | 1.016 |
| Sca | THYCA-Somatic | 0.179 | 0.029 | 0.177 |
| Chi | THYCA-Somatic | 0.221 | 0.014 | 0.015 |
| Tin | THYCA-Somatic | 0.000 | 0.022 | 0.013 |
| Wei | THYCA-Somatic | 0.078 | 0.000 | 0.000 |
| Hea | THYCA-Somatic | 0.108 | 0.256 | 0.168 |
| Sex | THYCA-Somatic | 0.151 | 0.010 | 0.059 |
| Psy | Psychological | 0.755 | 0.767 | 0.976 |
| FCR | Psychological | 0.407 | 0.294 | 0.090 |
| Lon | Psychological | 0.159 | 0.107 | 0.343 |
| RW | Psychological | 0.203 | 0.409 | 0.376 |
| Sal | RAI-Somatic | 0.000 | 0.277 | 0.256 |
| Nec | RAI-Somatic | 0.054 | 0.381 | 0.458 |
| Tas | RAI-Somatic | 0.052 | 0.322 | 0.457 |
| Nau | RAI-Somatic | 0.006 | 0.530 | 0.519 |

*Bridge strength represents the sum of absolute edge weights connecting a node to nodes in other clusters.*

*T0: Pre-treatment; T1: 48h post-treatment; T2: 1 week post-discharge.*

**Table S4. Expected influence centrality across three timepoints**

| **Node** | **Cluster** | **T0** | **T1** | **T2** |
| --- | --- | --- | --- | --- |
| NM | THYCA-Somatic | 1.509 | -0.420 | -0.067 |
| Sen | THYCA-Somatic | 0.105 | -1.123 | -1.008 |
| Voi | THYCA-Somatic | -1.003 | -0.165 | 0.230 |
| Con | THYCA-Somatic | 0.909 | -0.352 | 0.413 |
| Sym | THYCA-Somatic | -0.005 | -0.731 | -0.987 |
| TM | THYCA-Somatic | -0.772 | 1.470 | 1.387 |
| Sca | THYCA-Somatic | -0.453 | -1.053 | -0.518 |
| Chi | THYCA-Somatic | 1.417 | -0.859 | -1.007 |
| Tin | THYCA-Somatic | -0.218 | -1.047 | -1.272 |
| Wei | THYCA-Somatic | -0.238 | -1.324 | -1.387 |
| Hea | THYCA-Somatic | -0.484 | -0.588 | -0.708 |
| Sex | THYCA-Somatic | -0.574 | -1.091 | -1.231 |
| Psy | Psychological | 1.776 | 2.032 | 2.298 |
| FCR | Psychological | 1.333 | 1.025 | 0.506 |
| Lon | Psychological | 0.609 | 0.253 | 0.588 |
| RW | Psychological | 0.772 | 0.863 | 0.597 |
| Sal | RAI-Somatic | -1.166 | 0.961 | 0.676 |
| Nec | RAI-Somatic | -1.280 | 0.727 | 0.716 |
| Tas | RAI-Somatic | -1.056 | 0.586 | 0.889 |
| Nau | RAI-Somatic | -1.179 | 0.835 | -0.116 |

*Expected influence preserves edge weight signs. Positive values indicate activating influence; negative values indicate inhibitory influence.*

*T0: Pre-treatment; T1: 48h post-treatment; T2: 1 week post-discharge.*

**Table S5. Bridge expected influence centrality across three timepoints**

| **Node** | **Cluster** | **T0** | **T1** | **T2** |
| --- | --- | --- | --- | --- |
| NM | THYCA-Somatic | 0.254 | 0.151 | 0.202 |
| Sen | THYCA-Somatic | 0.000 | 0.000 | 0.016 |
| Voi | THYCA-Somatic | 0.000 | 0.331 | 0.535 |
| Con | THYCA-Somatic | 0.173 | 0.181 | 0.305 |
| Sym | THYCA-Somatic | 0.332 | 0.189 | 0.081 |
| TM | THYCA-Somatic | 0.107 | 1.017 | 1.016 |
| Sca | THYCA-Somatic | 0.179 | 0.029 | 0.177 |
| Chi | THYCA-Somatic | 0.182 | 0.014 | 0.015 |
| Tin | THYCA-Somatic | 0.000 | 0.022 | 0.013 |
| Wei | THYCA-Somatic | 0.078 | 0.000 | 0.000 |
| Hea | THYCA-Somatic | 0.062 | 0.256 | 0.168 |
| Sex | THYCA-Somatic | 0.149 | -0.010 | 0.059 |
| Psy | Psychological | 0.734 | 0.767 | 0.976 |
| FCR | Psychological | 0.407 | 0.294 | 0.090 |
| Lon | Psychological | 0.159 | 0.107 | 0.343 |
| RW | Psychological | 0.203 | 0.409 | 0.376 |
| Sal | RAI-Somatic | 0.000 | 0.277 | 0.256 |
| Nec | RAI-Somatic | -0.054 | 0.365 | 0.458 |
| Tas | RAI-Somatic | 0.052 | 0.303 | 0.457 |
| Nau | RAI-Somatic | -0.006 | 0.530 | 0.519 |

*Bridge expected influence represents the sum of edge weights (with signs) connecting a node to nodes in other clusters.*

*T0: Pre-treatment; T1: 48h post-treatment; T2: 1 week post-discharge.*

**Table S6. Predictability (R²) values across three timepoints**

| **Node** | **Cluster** | **T0** | **T1** | **T2** | **Mean** |
| --- | --- | --- | --- | --- | --- |
| NM | THYCA-Somatic | 0.703 | 0.156 | 0.255 | 0.371 |
| Sen | THYCA-Somatic | 0.386 | 0.000 | 0.036 | 0.141 |
| Voi | THYCA-Somatic | 0.000 | 0.454 | 0.551 | 0.335 |
| Con | THYCA-Somatic | 0.624 | 0.177 | 0.358 | 0.386 |
| Sym | THYCA-Somatic | 0.346 | 0.090 | 0.000 | 0.145 |
| TM | THYCA-Somatic | 0.064 | 0.782 | 0.788 | 0.545 |
| Sca | THYCA-Somatic | 0.102 | 0.000 | 0.091 | 0.064 |
| Chi | THYCA-Somatic | 0.651 | 0.000 | 0.024 | 0.225 |
| Tin | THYCA-Somatic | 0.281 | 0.000 | 0.000 | 0.094 |
| Wei | THYCA-Somatic | 0.184 | 0.000 | 0.000 | 0.061 |
| Hea | THYCA-Somatic | 0.102 | 0.124 | 0.000 | 0.075 |
| Sex | THYCA-Somatic | 0.122 | 0.000 | 0.000 | 0.041 |
| Psy | Psychological | 0.786 | 0.706 | 0.759 | 0.750 |
| FCR | Psychological | 0.718 | 0.674 | 0.632 | 0.675 |
| Lon | Psychological | 0.598 | 0.531 | 0.565 | 0.565 |
| RW | Psychological | 0.676 | 0.615 | 0.588 | 0.626 |
| Sal | RAI-Somatic | 0.000 | 0.742 | 0.744 | 0.495 |
| Nec | RAI-Somatic | 0.000 | 0.704 | 0.743 | 0.482 |
| Tas | RAI-Somatic | 0.000 | 0.701 | 0.754 | 0.485 |
| Nau | RAI-Somatic | 0.000 | 0.641 | 0.286 | 0.309 |

*Predictability (R²) represents the proportion of variance in each node that can be explained by its neighbors in the network.*

*T0: Pre-treatment; T1: 48h post-treatment; T2: 1 week post-discharge.*

**Table S7. Centrality stability coefficients (CS-coefficients) across three timepoints**

| **Centrality Index** | **T0** | **T1** | **T2** |
| --- | --- | --- | --- |
| Strength | 0.75 | 0.75 | 0.75 |
| Expected Influence | 0.75 | 0.75 | 0.75 |

*CS-coefficient represents the maximum proportion of cases that can be dropped while maintaining a correlation of at least 0.7 with the original centrality indices.*

*Values ≥0.5 indicate good stability; values ≥0.25 indicate acceptable stability.*

*T0: Pre-treatment; T1: 48h post-treatment; T2: 1 week post-discharge.*

| **Table S8. Zero-order Pearson correlations among five RAI treatment-related symptom items and THYCA-QoL throat/mouth scale across three timepoints** | | | | | | | |
| --- | --- | --- | --- | --- | --- | --- | --- |
| **T0 (Pre-treatment)** |  |  |  |  |  |  |  |
| Item | Description | Sal | Nec | Tas | Nau | RW |  |
| Sal | Salivary symptoms | — |  |  |  |  |  |
| Nec | Neck symptoms | -0.030 | — |  |  |  |  |
| Tas | Taste alterations | -0.038 | 0.001 | — |  |  |  |
| Nau | Nausea | 0.054 | -0.014 | -0.042 | — |  |  |
| RW | Radiation worry | 0.024 | -0.074 | 0.038 | -0.042 | — |  |
|  |  |  |  |  |  |  |  |
| **T1 (48h post-treatment)** |  |  |  |  |  |  |  |
| Item | Description | Sal | Nec | Tas | Nau | RW |  |
| Sal | Salivary symptoms | — |  |  |  |  |  |
| Nec | Neck symptoms | 0.779 | — |  |  |  |  |
| Tas | Taste alterations | 0.792 | 0.733 | — |  |  |  |
| Nau | Nausea | 0.748 | 0.726 | 0.716 | — |  |  |
| RW | Radiation worry | 0.607 | 0.545 | 0.571 | 0.627 | — |  |
|  |  |  |  |  |  |  |  |
| **T2 (1 week post-discharge)** |  |  |  |  |  |  |  |
| Item | Description | Sal | Nec | Tas | Nau | RW |  |
| Sal | Salivary symptoms | — |  |  |  |  |  |
| Nec | Neck symptoms | 0.804 | — |  |  |  |  |
| Tas | Taste alterations | 0.818 | 0.784 | — |  |  |  |
| Nau | Nausea | 0.413 | 0.430 | 0.479 | — |  |  |
| RW | Radiation worry | 0.544 | 0.581 | 0.607 | 0.468 | — |  |
|  |  |  |  |  |  |  |  |
| **THYCA-QoL Throat/Mouth scale correlations with RAI items** | | | | | | |  |
|  |  | Sal | Nec | Tas | Nau | RW |  |
| T0 | ThroatMouth (TM) | 0.059 | -0.004 | 0.002 | -0.026 | 0.188 |  |
| T1 | ThroatMouth (TM) | 0.785 | 0.784 | 0.779 | 0.774 | 0.664 |  |
| T2 | ThroatMouth (TM) | 0.771 | 0.808 | 0.809 | 0.517 | 0.681 |  |
| *Sal = salivary symptoms; Nec = neck symptoms; Tas = taste alterations; Nau = nausea; RW = radiation worry; TM = throat/mouth symptoms. T0: Pre-treatment; T1: 48h post-treatment; T2: 1 week post-discharge. At T0, near-zero correlations confirm construct independence prior to RAI exposure. Elevated correlations at T1 reflect concurrent symptom onset from shared RAI tissue effects rather than construct redundancy. Differential attenuation of nausea correlations at T2 is consistent with its faster clinical resolution.* | | | | | | | |
