# Supplementary figures and images for: Longitudinal dynamics of symptom networks in patients with differentiated thyroid cancer undergoing radioactive iodine therapy: a prospective cohort study
Source: Front Oncol. 2026 Apr 30;16:1776771. doi: 10.3389/fonc.2026.1776771 (PMC13171378; doi:10.3389/fonc.2026.1776771)

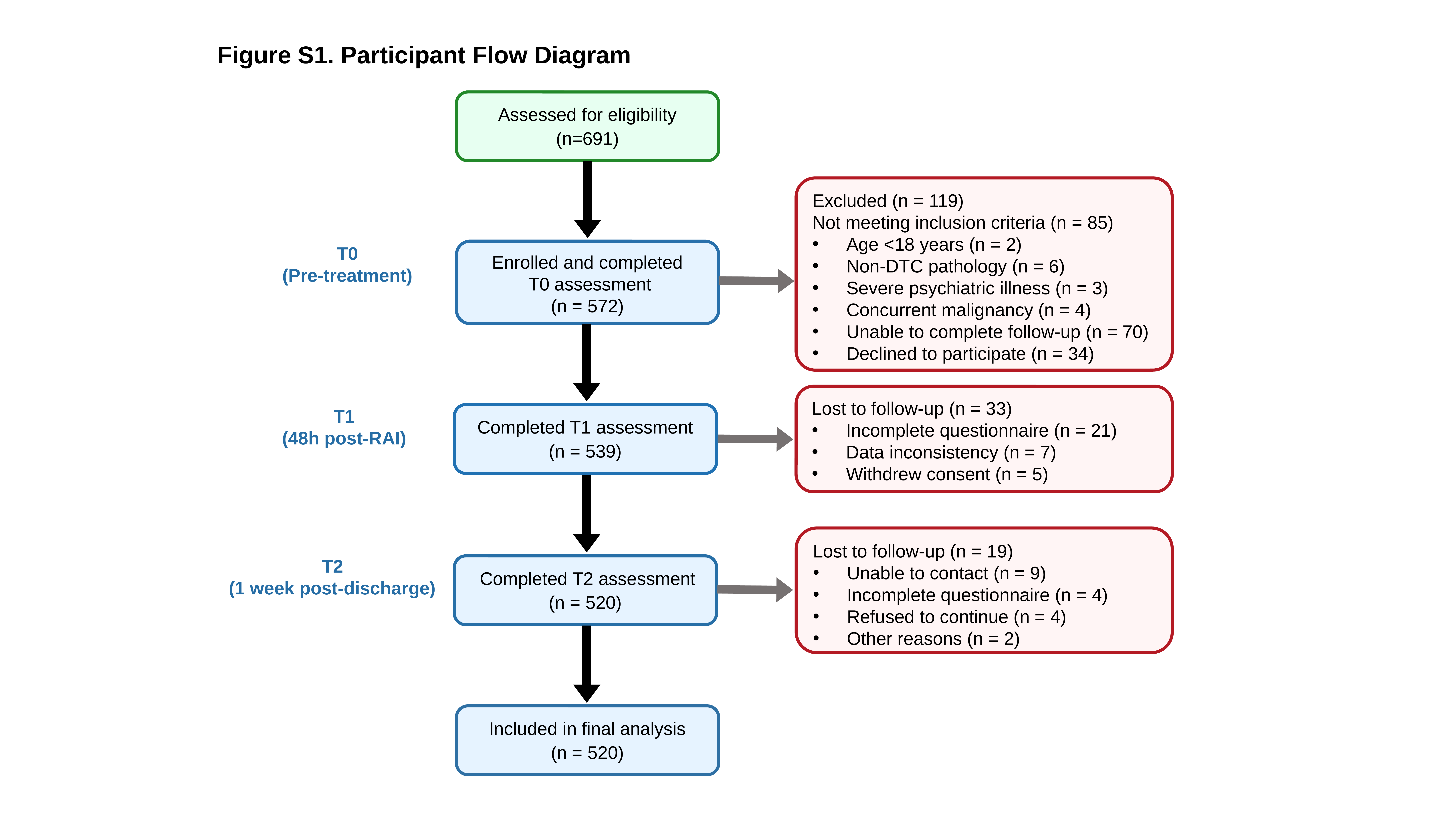

Supplement: Supplementary Figure 1 — Participant flow diagram showing enrollment, follow-up, and attrition at each assessment timepoint. [file Image1.tiff]

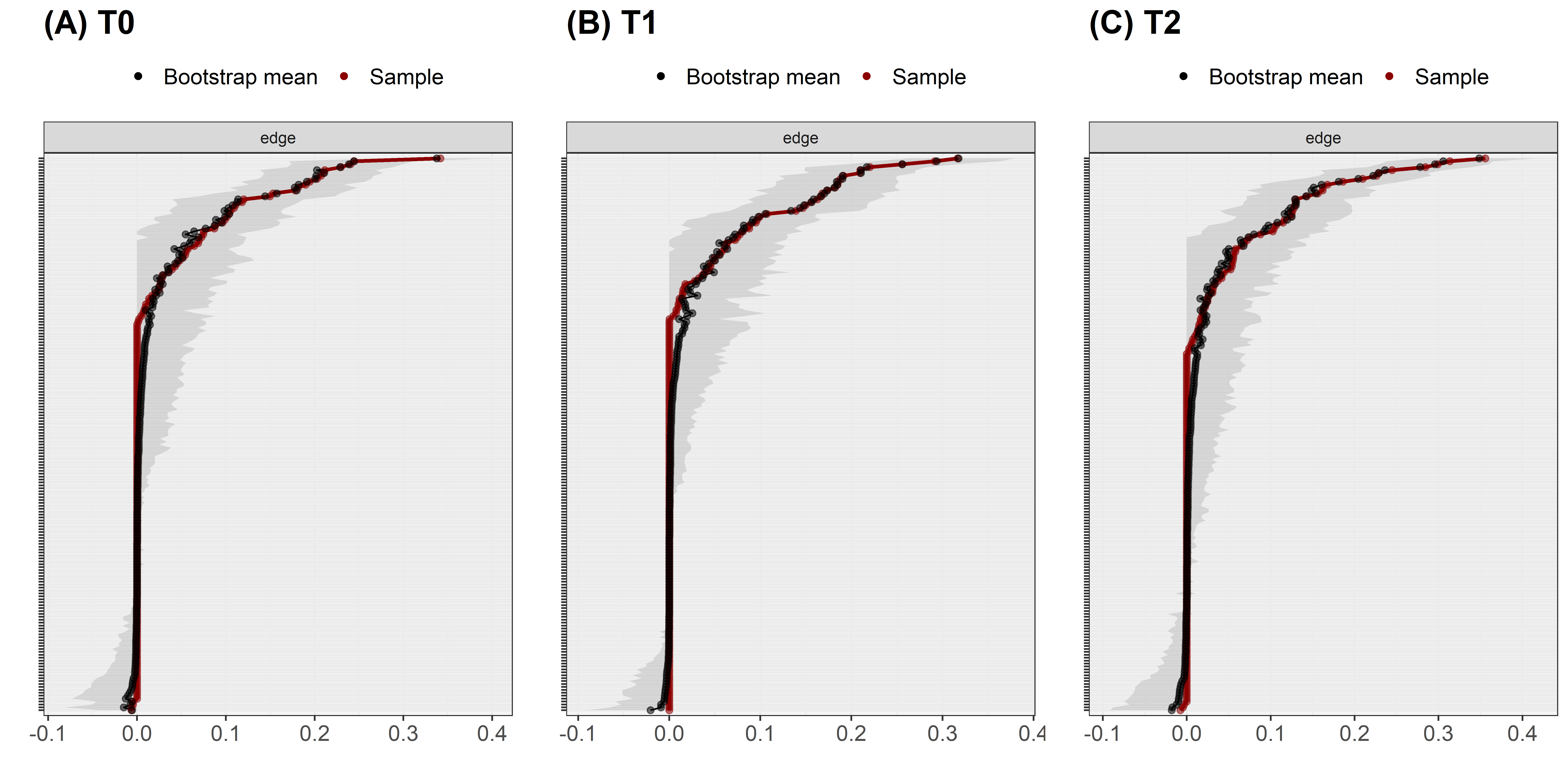

Supplement: Supplementary Figure 2 — Edge weight accuracy (bootstrap 95% CI) at T0, T1, and T2. [file Image2.tiff]

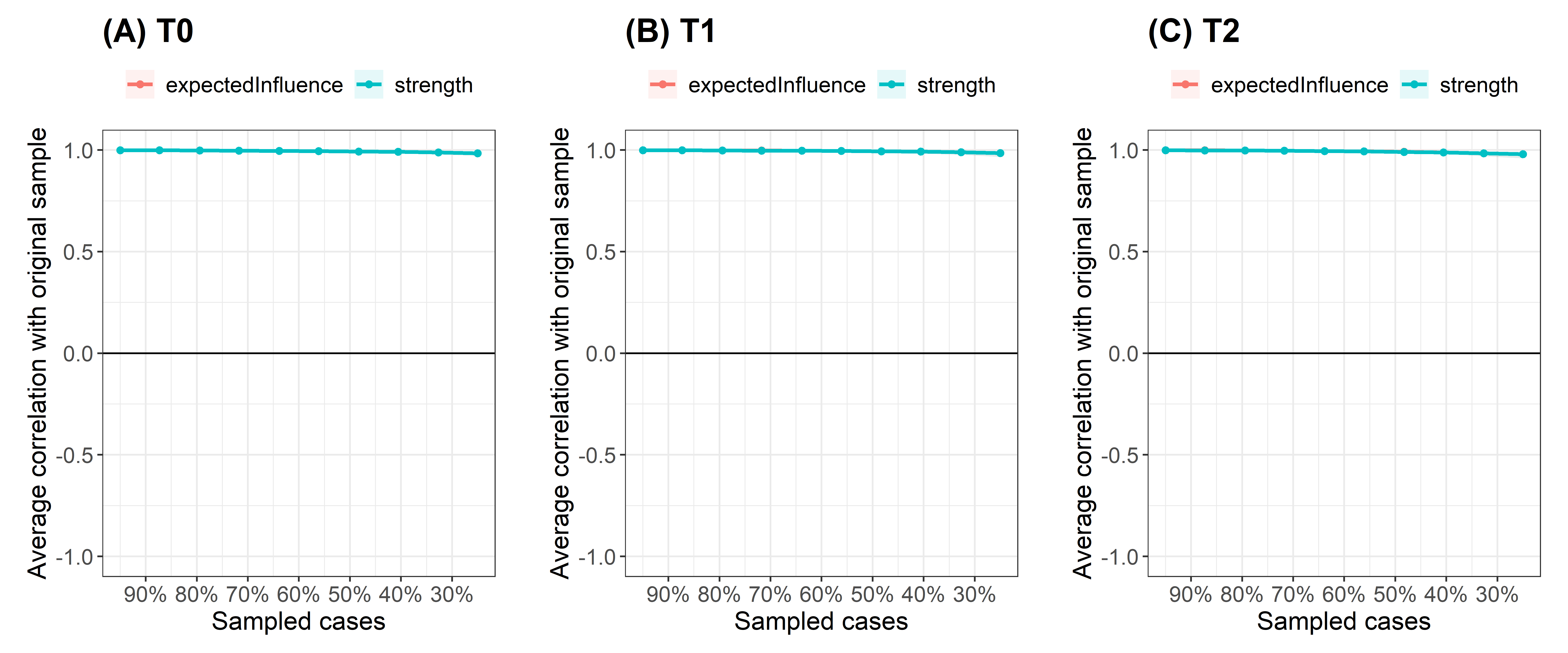

Supplement: Supplementary Figure 3 — Centrality stability (case-dropping bootstrap) at T0, T1, and T2. [file Image3.tiff]

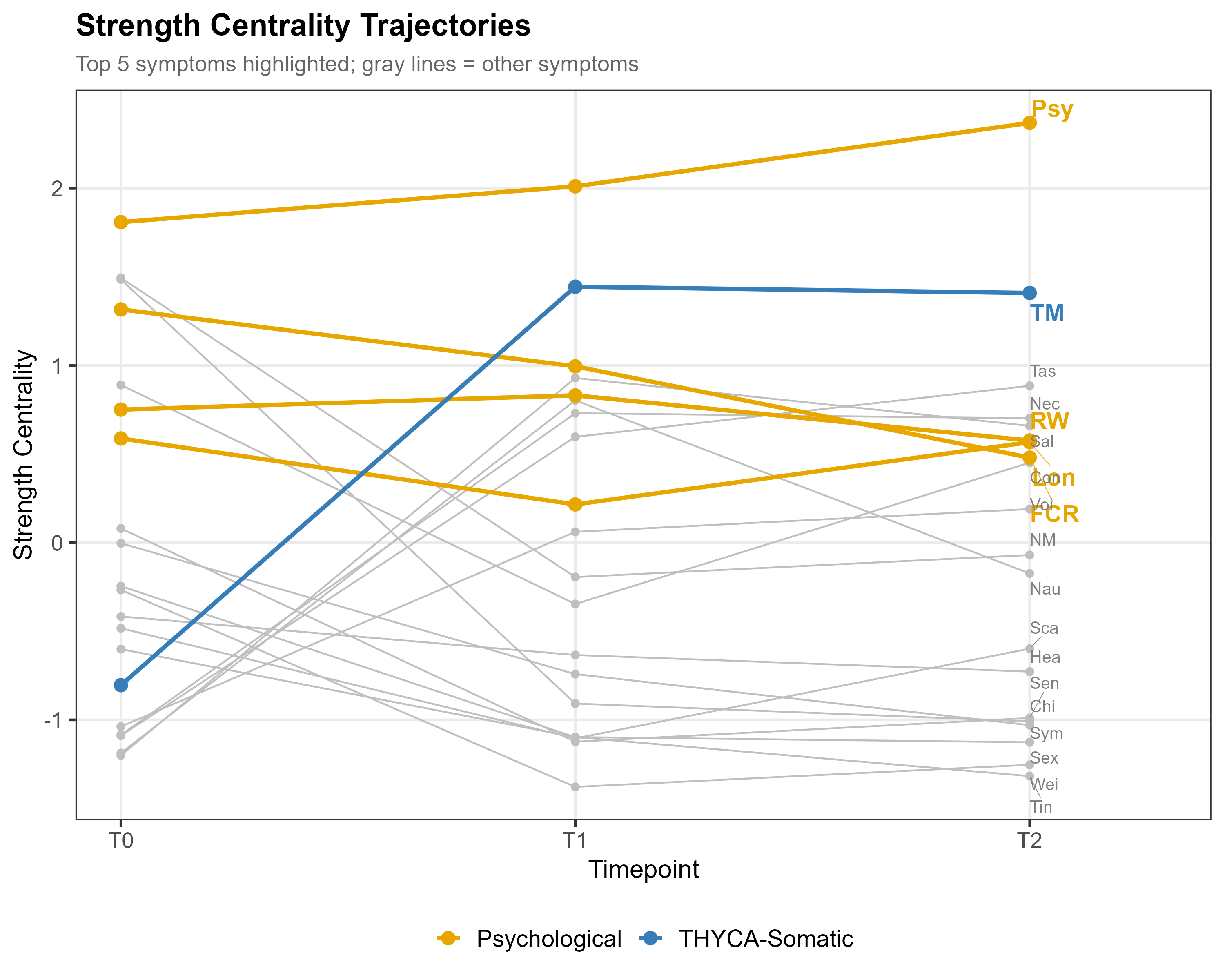

Supplement: Supplementary Figure 4 — Strength centrality trajectories across timepoints. [file Image4.tiff]

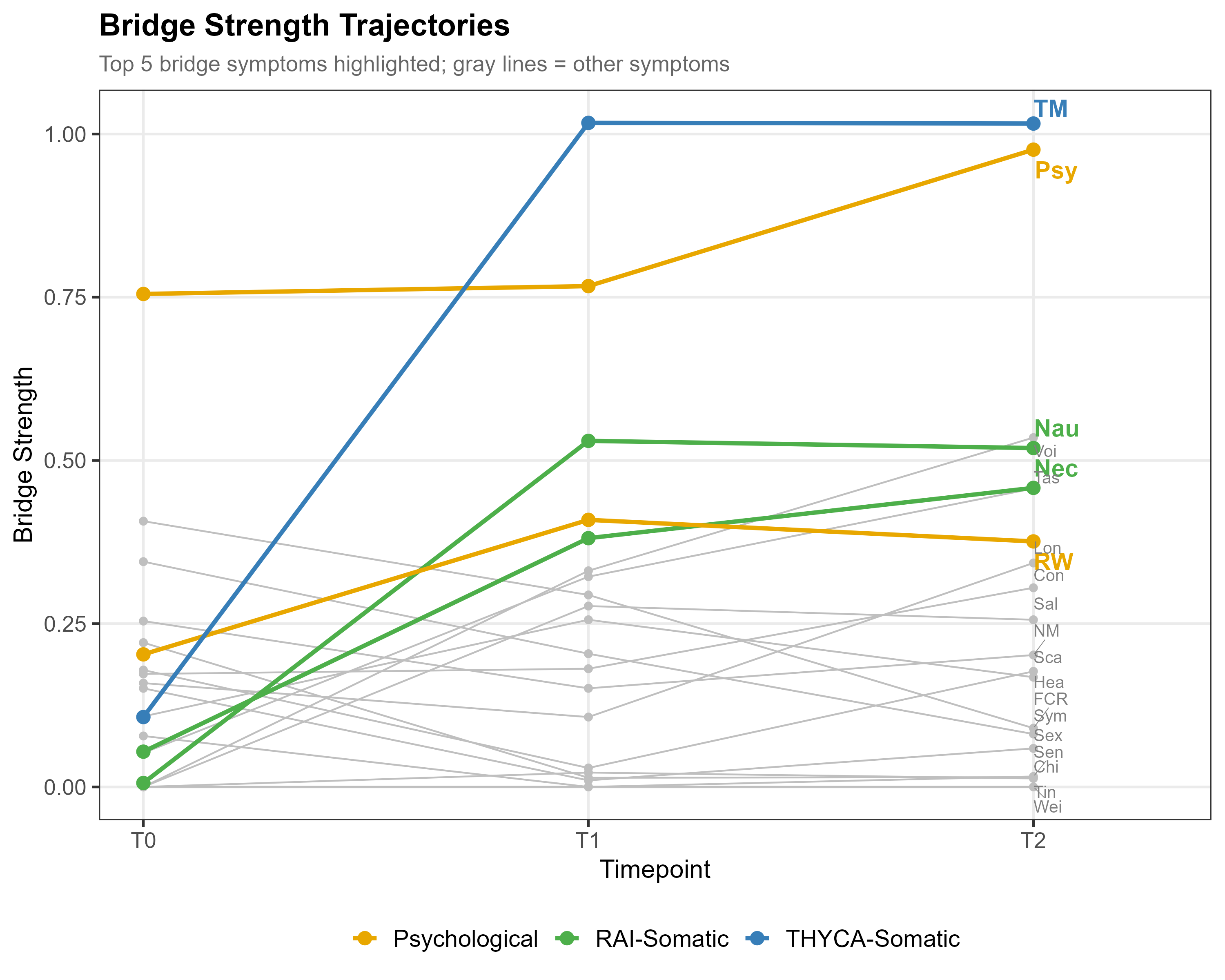

Supplement: Supplementary Figure 5 — Bridge strength centrality trajectories across timepoints. [file Image5.tiff]
